# Negative Impact of *Pseudomonas aeruginosa* Y12 on Its Host *Musca domestica*

**DOI:** 10.3389/fmicb.2021.691158

**Published:** 2021-07-14

**Authors:** Qian Zhang, Shumin Wang, Xinyu Zhang, Ruiling Zhang, Zhong Zhang

**Affiliations:** ^1^Collaborative Innovation Center for the Origin and Control of Emerging Infectious Diseases, Shandong First Medical University (Shandong Academy of Medical Sciences), Tai’an, China; ^2^School of Basic Medical Sciences, Shandong First Medical University (Shandong Academy of Medical Sciences), Tai’an, China

**Keywords:** biodegradation, *Pseudomonas aeruginosa*, gut bacteria, dysbacteriosis, multiple interactions, *Musca domestica* (L), housefly larvae

## Abstract

High concentrations of *Pseudomonas aeruginosa* Y12 significantly inhibit the development of housefly larvae and accelerate larvae death. In this study, the dynamic distribution of the gut microbiota of housefly larvae fed different concentrations of *P. aeruginosa* Y12 was investigated. Compared with low-concentration *P. aeruginosa* diets, orally administered high-concentration *P. aeruginosa* diets caused higher mortality and had a greater impact on the community structure and interaction network of intestinal flora in housefly larvae. The bacterial community of the gut microbiota in housefly larvae was reconstructed in 4 days. Bacterial abundance and diversity were significantly reduced in housefly larvae fed high concentrations of *P. aeruginosa*. With the growth of larvae, the relative abundances of *Providencia*, *Proteus*, *Myroides*, *Klebsiella*, and *Alcaligenes* increased significantly in housefly larvae fed with high concentrations of *P. aeruginosa*, while the relative abundances of *Bordetella*, *Enterobacter*, *Morganella*, *Ochrobactrum*, *Alcaligenaceae*, and *Empedobacter* were significantly reduced. To analyze the role of the gut microorganisms played on housefly development, a total of 10 cultivable bacterial species belonging to 9 genera were isolated from the intestine of housefly larvae among which *Enterobacter hormaechei*, *Klebsiella pneumoniae*, *Enterobacter cloacae*, *Lysinibacillus fusiformis*, and *Bacillus safensis* promoted the growth of larvae through feeding experiments. This study is the first to analyze the influence of high concentrations of *P. aeruginosa* on the gut microbiota of houseflies. Our study provides a basis for exploring the pathogenic mechanism of high concentrations of *P. aeruginosa* Y12 in houseflies.

## Introduction

Housefly (*Musca domestica*) is one of the most widespread insects in the environment. Houseflies ingest various organic wastes and have been widely applied in environmental waste deposal areas (Raksasat et al., 2020). Housefly larvae can be used as protein-rich feed ingredients in the field of cattle breeding, providing nutrition for the growth of farmed animals (Van Huis, 2013). The larvae of green bottle fly *Lucilia sericata* (Diptera: Calliphoridae) have also been applied in the healing of chronic wounds, called maggot therapy (Stadler, 2020). Maggot therapy enabled faster tissue growth and introduced a smaller wound surface area than traditional wound management (King, 2020). However, pathogen infections would inhibit the development of housefly larvae and threatened their further applications in waste biodegradation and sustainable animal feed production (Wang et al., 2020).

Houseflies harbor a wide variety of microorganisms in their intestinal tract providing their host with physiological advantages and helping them defend against pathogen invasion (Dillon and Dillon, 2004; Engel and Moran, 2013; Leclercq et al., 2019). Gut bacteria have also been reported to be involved in various biological functions in their host, contributing to nitrogen and carbon metabolism (Ben-Yosef et al., 2014) and providing nutritional benefits to the host (Douglas, 1998). In the fruit fly *Rhagoletis pomonella* (Diptera: Tephritidae), gut bacteria participate in the degradation of plant-derived toxic compounds, which provide proteins for host development (Lauzon et al., 2003). In Mediterranean fruit fly *Ceratitis capitata* (Diptera: Tephritidae), gut bacterial communities enhance host copulatory success, defend their host against deleterious bacterial infections and affect host longevity (Behar et al., 2008). The imbalance of the intestinal flora affects the development of insects and leads to various diseases (Ben-Yosef et al., 2008). It has been reported that feeding probiotics (*Klebsiella oxytoca*) significantly improved males’ sexual competitiveness and prolonged their survival in Mediterranean fruit fly *C. capitata* (Ben Ami et al., 2010; Gavriel et al., 2011). *Candidatus* Erwinia dacicola in olive fly adults (*Bactrocera oleae*) can turn essential amino acids and metabolize urea into available nitrogen sources, thus increasing egg production (Ben-Yosef et al., 2014). The symbiotic olive fly (*Bactrocera oleae*) larval bacterium *Erwinia dacicola* can neutralize the inhibitory effect of oleuropein (a kind of the principal phenolic glycoside in unripe olives) to promote the development of larvae (Ben-Yosef et al., 2015). Metabolites of *Bacillus cereus* can lure massive adult oriental fruit flies *Bactrocera dorsalis*, providing good information for the development of bacterial biocontrol agents and the production of insecticides (Wang H. et al., 2014).

The dominant intestinal flora of housefly larvae gradually forms during the development process. *Providencia*, *Proteus*, and *Kurthia* were commonly the dominant genera in most species, followed by *Pseudomonas, Klebsiella, Alcaligenes*, and *Myroide* (Su et al., 2010). The structure of intestinal bacterial communities in housefly larvae can be influenced by different diets (Xue et al., 2019). Studies have revealed that the houseflies form a relatively stable internal bacterial microbial community according to geographical location and habitat, and alteration of the bacterial community structure would lead to increased mortality (Park et al., 2019).

*Pseudomonas aeruginosa* is a ubiquitous bacterium found in soil and water. It is an opportunistic pathogen and is associated with significant mortality in animals and insects (Osborn et al., 2002; Hilbi et al., 2007; Kostylev et al., 2019). In the Mediterranean fruit fly (*C. capitata*), high concentrations of *P. aeruginosa* in the gut can reduce the longevity of the host (Behar et al., 2008). Our previous study reported a *P. aeruginosa* strain Y12 and revealed that low concentrations of *P. aeruginosa* Y12 can protect housefly larvae from *Beauveria bassiana* infections through the production of antifungal compounds, while high concentrations of *P. aeruginosa* in the habitat of housefly larvae significantly inhibited the development of housefly larvae (Wang et al., 2020). Therefore, high concentrations of *P. aeruginosa* in the habitats can be life-threatening to the housefly larvae. For effective development and utilization of housefly larvae resource, the pathogenesis of *P. aeruginosa* needs further investigation. However, there are still no studies explaining the mechanism by which *P. aeruginosa* inhibits the development of its host.

In this study, we analyzed the impact of *P. aeruginosa* Y12 on the microbial community structure and development of housefly larvae. The changes in the composition of the intestinal flora of housefly larvae fed different concentrations of *P. aeruginosa* Y12 were analyzed through 16S rRNA gene sequencing technology, and we found that both bacterial diversity and richness in the intestine of housefly larvae fed a high concentration of *P. aeruginosa* Y12 were significantly reduced. Based on our study, we assume that alteration of the housefly intestinal flora induced by high concentration *P. aeruginosa* Y12 infections could be an important reason that inhibit the growth and development of house flies.

## Materials and Methods

### Materials

A housefly (*Musca domestica*) colony was reared and maintained in the Laboratory of Vector and Insect Diseases of Shandong First Medical University since 2005. The housefly adults were fed with brown sugar and water, and the larvae were fed with wet wheat bran and milk powder [Wheat bran(g): water(mL): milk powder(g) = 1:1:0.4]. They were raised in an artificial climate incubator with a temperature of 25 ± 1°C and 70% relative humidity with a photoperiod of 12/12 h (L/D). *P. aeruginosa* Y12 was isolated from the larval intestine of this strain.

### Insect Rearing and Experiment Design Involving *P. aeruginosa*

*Pseudomonas aeruginosa* Y12 was inoculated into freshly prepared LB liquid medium and placed in a constant temperature shaker. After shaking at 110 rpm for 24 h at 37°C, the concentration of *P. aeruginosa* was 4.63 × 10^8^ cfu/mL, which was used as a high concentration of *P. aeruginosa*. Five milliliters of *P. aeruginosa* stock solution was diluted stepwise with sterile water, and finally, the *P. aeruginosa* stock solution (Ya, 4.63 × 10^8^ cfu/mL) and the stock solution diluted 10^2^ (Yb, 4.63 × 10^6^ cfu/mL), 10^4^ (Yc, 4.63 × 10^4^ cfu/mL), 10^6^ (Yd, 4.63 × 10^2^ cfu/mL), and 10^8^ (Ye, 4.63 cfu/mL) fold were used for feeding experiments. To analyze the influence of the fermentation broth of *P. aeruginosa* on the growth of housefly larvae, *P. aeruginosa* stock solution (Ya) was centrifuged at 6,000 × *g* for 5 min to obtain the fermentation broth supernatant.

Ya, Yb, Yc, Yd, and Ye *P. aeruginosa* suspensions were used as the experimental groups, and sterile water (Wa) and the fermentation broth supernatant were used as control groups. Diluted *P. aeruginosa* culture suspensions was mixed with sterilized wheat bran at a ratio of 2:1. A 10 mL centrifuge tube with a small hole on the top was used to ensure air permeability, an equal amount of wheat bran was placed in each centrifuge tube, and then 10 normal-breeding, good-growing, uniform-sized 1-day-old larvae were added for a total of 6 groups with 150 per group. A piece of gauze was put between the tube and the lid to prevent the larvae from escaping. Each group was set up in 3 repetitions. They were placed in an artificial climate incubator with a temperature of 25 ± 1°C, relative humidity of 70 ± 5%, and a photoperiod of 16/8 h (L/D).

In each group of larvae reared above, from the first day after rearing, 3 larvae were removed from each tube at the same time every day to record the length and weight of the larvae and observe the results of biological indicators such as larval pupation rate and eclosion rate in each tube.

Surface debris was removed from the larvae, which were then placed in a 1.5 mL centrifuge tube containing 75% alcohol, soaked and disinfected for 10–15 min, and then rinsed with sterile deionized water 3 times to remove the bacteria attached to the surface. This disinfection and rinsing process was repeated 3 times. After strict body surface disinfection, the housefly larvae samples were stored at −80°C and sent for high-throughput sequencing. The larvae taken out of different treatment groups and control groups each time were used as a sampling unit, and each sampling unit had 3 replicates.

### Confrontation Experiment Between *P. aeruginosa* and Cultivable Bacteria in the Gut of Houseflies

Cultivable bacteria were isolated through the traditional separation culture method. The housefly samples were soaked in 75% alcohol for 10 min and cleaned three times with sterile ddH_2_O to disinfect the body surface. Completely grind the sample with an automatic grinder, add 100 μl sterile water and thoroughly mix, take 50 μL mixture and diluted to 10^2^, 10^4^, 10^6^ fold, and 100 μL of each mixture was evenly coated on nutrient agar medium, placed in a constant temperature incubator for 24 h, and the bacteria were incubated at 37°C until colony was formed. According to the differences of bacteria morphology, color, size, transparency, and other characteristics, single colony was selected and inoculated into a new nutrient agar medium. All the experimental operations were strictly aseptic. The isolated bacteria and *P. aeruginosa* were inoculated in LB liquid medium and cultured at 37°C for 24 h (at 110 rpm). *P. aeruginosa* culture was inoculated on half of a nutrient agar plate using the spread plate method with a sterile cotton swab, and the opposite side of the agar plate was used as a negative control. Then, two 6-mm-diameter sterile filter papers were symmetrically placed on the two sides of agar medium, and 10 μL of the isolated cultivable gut bacteria, including *Enterobacter hormaechei, Klebsiella pneumoniae, Pseudomonas aeruginosa, Acinetobacter bereziniae, Providencia stuartii, Enterobacter cloacae, Lactococcus lactis, Lysinibacillus fusiformis, Providencia vermicola*, and *Bacillus safensis*, were added to the filter papers. The plates were cultured at 28°C for 48 h. The colony sizes of the different isolated bacteria were measured to evaluate the interactions between *P. aeruginosa* and the different cultivable gut bacteria. The experiments were conducted with three independent biological replications.

### The Influence of the Isolated Bacteria on the Growth of Housefly Larvae

The intestinal bacterial culture of housefly larvae was used as the experimental group, LB culture stock was used as a negative control group, sterile water was used as a blank control group, and bacterial culture solution was mixed with sterilized wheat bran at a ratio of 2:1. The mixed wheat bran was put into 10 mL centrifuge tubes with small holes on the top. A total of 10 1-day-old larvae were placed in each centrifuge tube. Culture chambers were adjusted to 70 ± 5% RH and a 12/12 h L/D photoperiod at 25 ± 1°C. The body length, weight, pupation rate, and eclosion rate of the housefly larvae were recorded every day at the same time.

### Extraction of the Intestine DNA

All the samples were surface-disinfected individually with 70 and 90% (vol/vol) ethanol solution for 1 min, respectively, and then rinsed three times with sterile water in order to remove bacteria from the larvae surface. The abdomen was dissected with sterile forceps and dissecting needle in clean bench, and the whole digestive tract was removed and transferred into a 1.5 ml centrifuge tubes. Then the tubes were filled with 100 μL double-distilled water and ceramic beads (0.1 mm) for the subsequent DNA extraction. Intestine samples were homogenized in tissue Lyser (Qiagen, Hilden, Germany) followed by genomic DNA extraction using the Wizard Genomic DNA purification kit (Promega; A1120). Quantification of total DNA was performed after each DNA extraction using NanoDrop 2000 spectrophotometer (Thermo Fisher Scientific, Waltham, MA, United States) and 2% agarose gel electrophoresis, respectively. Extracted DNA was stored at −20°C until further processing.

### PCR Amplification, Illumina MiSeq Sequencing and Bioinformatics Analysis

The hypervariable V3-V4 region of the bacterial 16S rRNA gene was amplified with the primers 341F (5′-CCTAYGG GRBGCASCAG-3′) and 806R (5′-GGACTACNNGGGTATCT AAT-3′) (An improved dual-indexing approach for multiplexed 16S rRNA gene sequencing on the Illumina MiSeq platform, Shandong KeGene Science & Technology Co., Ltd.). Twenty-microliter PCR mixtures were set up with 4 μl 5 × FastPfu buffer, 2 μl dNTPs (2.5 mM), 0.8 μl each primer, 0.4 μl FastPfu polymerase, and template DNA (10 ng). Reactions proceeded in a GeneAmp 9700 (ABI) thermocycler with 95°C for 5 min; 27 cycles of denaturation at 95°C for 30 s, annealing at 55°C for 30 s, and elongation at 72°C for 45 s, followed by an additional elongation at 72°C for 10 min; and a dissociation stage at the end of the run.

PCR products were detected by 2% agarose gel electrophoresis and purified using the QIAquick gel extraction kit (Qiagen). Library pools were constructed with equal amounts of each PCR product by using the TruSeq Nano DNA LT sample prep kit (Illumina), which was amplified through the paired-end sequenced on the Illumina MiSeq PE300 platform.

The quality control of the original data was carried out using Trimmomatic v0.39 software^1^. Based on the overlap (minimum: 10 bp) between PE reads after quality control, PE reads were assembled using Flash v1.2.11 software (FLASH: fast length adjustment of short reads to improve genome assemblies). QIIME v1.9.1 software (QIIME allows analysis of high-throughput community sequencing data) was adopted for processing, and VSEARCH v2.14.1 software (VSEARCH: a versatile open-source tool for metagenomics) was used for detecting chimera sequences.

Based on a sequence similarity level of 97%, the Uclust method in the QIIME software package was employed to perform OTU clustering analysis. Based on the Silva reference database (Release138), taxonomic annotations were made for the OTUs of each sample. The Shannon, Simpson, Chao1, and ace indices of microbial communities were calculated by Mothur^2^, respectively. Heatmap is performed using R software. The common and unique OTUs were intuitively explained by Venn diagram. Principal Coordinates Analysis (PCoA) based on the Bray-Curtis dissimilarity and Unweighted Pair Group Method with Arithmetic Mean (UPGMA) tree based on unweighted UniFrac phylogenetic distances were used to determine the difference of beta diversity of bacterial communities in different samples.

Co-occurrence network analysis was based on following Molecular Ecological Network Analyses Pipeline (Deng et al., 2012). The OTU of all samples were retained for analysis, and the number of sequences was log-transformed and analyzed using a random matrix theory–based approach (Zhou et al., 2010). The edges (i.e., connections between taxa as OTUs) correspond to a significant (positive or negative) correlation between nodes (i.e., taxa as OTUs) (Dini-Andreote et al., 2014). The network was graphed by using Gephi. To identify potential keystone driver taxa based on differences in network interactions between the experimental group and control group microbiomes^3^ by using the NetShift method.

### Statistical Analysis

The experimental data were analyzed by Microsoft Excel 2010 and IBM SPSS Statistics 20 statistical software. All the data are given as mean ± SD. The effects of the isolated bacteria in the intestine on the body weight and length of housefly larvae were compared by using one-way ANOVA. Significance analysis was performed by Tukey’s test (*p* < *0.05*).

## Results

### Effect of *P. aeruginosa* Y12 on Housefly Larval Growth

**FIGURE 1:**
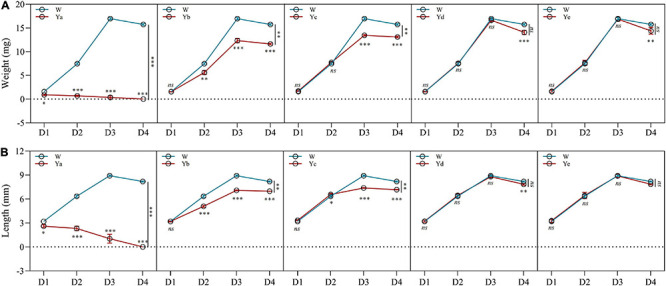
Effects of different dilutions of *P. aeruginosa* on the development of housefly larvae. **(A)** Body weight was significantly changed over time in housefly larvae with different dilutions of *P. aeruginosa*. **(B)** Body length was significantly changed over time in housefly larvae with different dilutions of *P. aeruginosa*. Wa, Ya, Yb, Yc, Yd, and Ye represent housefly larval samples fed diets with sterile water, *P. aeruginosa* stock solution, and the stock solution diluted 10^2^, 10^4^, 10^6^, and 10^8^ fold, respectively. Each treatment included three biological replicates. Data are represented as the mean ± SEM. Repeated measures ANOVA followed by Sidak correction was used for multiple comparisons. *p < 0.05, **p < 0.01, ***p < 0.001.

Different dilutions of *P. aeruginosa* Y12 were added to the housefly larval food, and the body weight, length, pupation rate, and eclosion rate of the housefly larvae fed different diets were analyzed. The body weight (4.71 and 74.66%) and length (22.38 and 83.75%) of the housefly larvae fed high-concentration *P. aeruginosa* (Ya and Yb) were significantly reduced compared to those of the control group (Wa). The development of housefly larvae fed Ya was significantly inhibited, as their pupation rate and eclosion rate were both reduced to 0%. As the *P. aeruginosa* concentration decreased in the larval diet (Yc), its inhibitory effect on the development of housefly larvae decreased (Figure 1). Compared to the control group (Wa), housefly larvae that fed on low-concentration *P. aeruginosa* (Yd and Ye) experienced no significant inhibitory effect in their development. Additionally, the fermentation broth supernatant of *P. aeruginosa* also exhibited little effect on the growth and development of housefly larvae (Supplementary Figure 1).

### DNA Sequence Data and Microbial Diversity Index Analysis

After raw data were quality filtered, 1918558 high-quality reads were obtained from the housefly larvae samples (NCBI BioProject ID: PRJNA725070). According to 99% sequence identity, a total of 17354 OTUs were found in all samples, among which 2501, 2339, 2517, 2627, 3218, and 4152 OTUs were separately obtained from samples in the Ya, Yb, Yc, Yd, Ye, and Wa groups, respectively. The community richness indices Chao1 and ACE showed that the abundances of the bacterial communities of housefly larvae fed with low concentrations of *P. aeruginosa* (Yd and Ye) were higher than those fed with high concentrations (Ya and Yb); The Shannon and Simpson community diversity indices indicated a higher bacterial community diversity in housefly larvae fed with low concentrations of *P. aeruginosa* (Yd and Ye) (Table 1).

**TABLE 1:** ACE, Chao1, Shannon, and Simpson indices of intestinal bacteria in different groups of houseflies.

**Sample ID**	**Reads**	**OTU**	**Biological index**				
			**Ace**	**Chao**	**Shannon**	**Simpson**	**Coverage**
Wa	62350	625	719.58 ± 121.88	689.00 ± 111.32	3.17 ± 0.56	0.14 ± 0.09	99.80%
Ya	69331	585	732.75 ± 147.22	710.00 ± 143.32	3.00 ± 0.44	0.14 ± 0.07	99.77%
Yb	67279	629	781.58 ± 158.39	750.83 ± 153.15	3.13 ± 0.46	0.14 ± 0.1	99.75%
Yc	66343	657	832.08 ± 116.04	790.00 ± 106.33	3.14 ± 0.18	0.12 ± 0.04	99.73%
Yd	62888	805	938.50 ± 117.45	907.92 ± 123.23	3.37 ± 0.33	0.10 ± 0.05	99.74%
Ye	59266	1038	1126.33 ± 144.63	1101.83 ± 140.71	3.53 ± 0.64	0.12 ± 0.08	99.78%

*Alpha diversity indices were analyzed using one-way ANOVA to characterize the microbial richness and diversity of the samples fed different dilutions of *P. aeruginosa*. Significance analysis was performed using Tukey’s test (*p* < 0.05). Wa, Ya, Yb, Yc, Yd, and Ye represent housefly larvae samples fed diets of sterile water, *P. aeruginosa* stock solution, and the stock solution diluted 10^2^, 10^4^, 10^6^, and 10^8^ fold, respectively. Each treatment included three biological replicates. The error estimate represents the standard error of the mean.*

**FIGURE 2:**
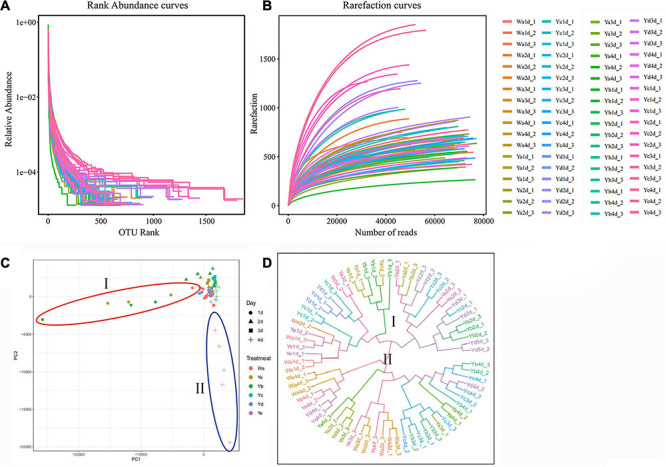
Species rarefaction analysis of sample sequences and differences in bacterial community structures among different groups. Species rank-abundance curve **(A)** and rarefaction curve **(B)** analysis of sample sequences. **(C)** Principal coordinate analysis (PCoA) of bacterial community structures of the different groups. **(D)** UPGMA tree analysis of evolving samples. Each symbol represents one sample of different dilutions of *P. aeruginosa.* Wa, Ya, Yb, Yc, Yd, and Ye represent housefly larvae samples fed diets with sterile water, *P. aeruginosa* stock solution, and the stock solution diluted 10^2^, 10^4^, 10^6^, and 10^8^ fold, respectively.

The species richness and evenness were further demonstrated through rank-abundance curves. The curves of housefly larvae samples fed with high concentrations of *P. aeruginosa* (Ya and Yb) tended to decrease very sharply, indicating their relatively low species richness (Figure 2A). However, the curves of samples fed with high concentrations of *P. aeruginosa* (Yd and Ye) were much less steep, indicating their higher species abundance. The rarefaction curves revealed that the depth of the sequencing data was sufficient for the data to represent the majority of species diversity and abundance (Figure 2B).

Principal coordinate analysis (PCoA) showed that the gut microbial communities of housefly larvae fed different concentrations of *P. aeruginosa* varied significantly and that the differences between the data for different sampling times were greater than the differences for various dilution rates of *P. aeruginosa* in the larval diet. In particular, in a short period (such as 1 and 2 d), larvae that fed on high-concentration *P. aeruginosa* were significantly different from others. However, after 3–4 days, bacterial components in larvae that fed on low-concentration *P. aeruginosa* exhibited higher differences than the control group (Figure 2C). UPGMA tree (unweighted pair group method with arithmetic mean tree) analysis further demonstrated the clustering of samples fed different concentrations of *P. aeruginosa* (Ya and Yb) (Figure 2D). Larvae samples fed high concentrations of *P. aeruginosa* (Ya and Yb) clustered together on the first day. However, after 3–4 days, samples fed low concentrations of *P. aeruginosa* (Yd and Ye) showed obvious clustering. The results indicated that feeding a low concentration of *P. aeruginosa* had less influence on the gut bacterial composition than feeding a high concentration of *P. aeruginosa.*

The differences in the gut community structures of samples fed different concentrations of *P. aeruginosa* were further supported by Venn diagrams that revealed OTUs shared between samples. Approximately 63 OTUs (25.8%) were shared among all samples. A total of 8 OTUs (83.28%) were shared by samples fed a high concentration of *P. aeruginosa* (Ya), while 26 OTUs (10.7%) were shared by those fed a low concentration of *P. aeruginosa* (Ye) (Supplementary Figure 2).

### Bacterial Community Composition of Housefly Larvae After Being Fed *P. aeruginosa* Y12

**FIGURE 3:**
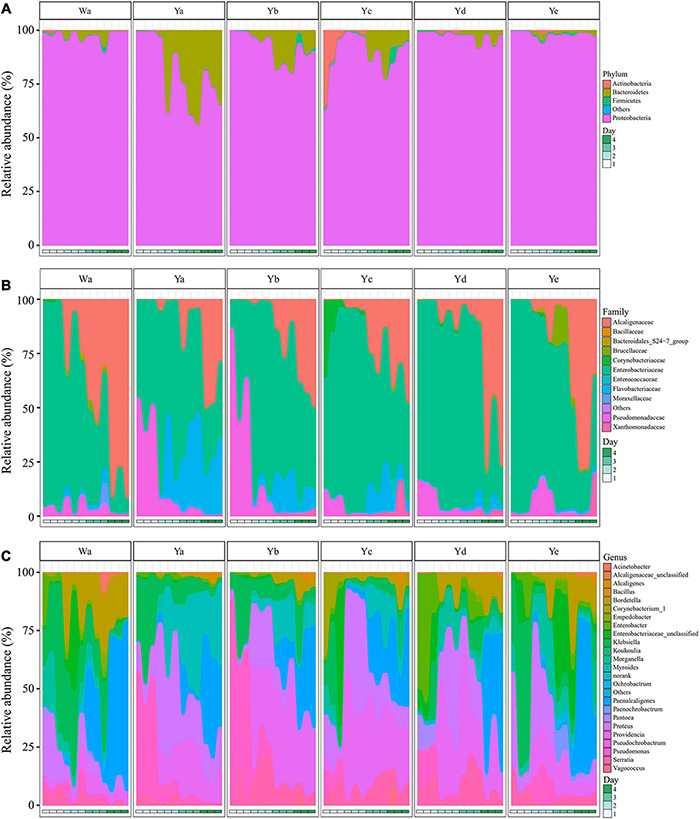
Relative abundances of bacterial components in samples of housefly larvae fed with different dilution concentrations of *P. aeruginosa*. **(A–C)** represent the relative abundances of bacteria at the phylum, family and genus classification levels, respectively. Wa, Ya, Yb, Yc, Yd, and Ye represent housefly larvae samples fed diets with sterile water, *P. aeruginosa* stock solution, and the stock solution diluted 10^2^, 10^4^, 10^6^, and 10^8^ fold, respectively.

The gut bacterial community compositions and structures were analyzed at different taxonomic levels. Proteobacteria was the predominant phylum in the 36 samples. A high abundance of Bacteroidetes was found in the Ya2–4d group, with less in the Yb2–4d and Yc3–4d groups, while Actinobacteria was abundant in the Yc1d group. After 4 days of feeding, the relative abundance of Proteobacteria accounted for 97.36% of the community in the control group, while it significantly decreased in the Ya4d, Yb4d, and Yc4d groups after feeding with a high concentration of *P. aeruginosa*. The relative abundance of Bacteroidetes significantly increased in the Ya (19.9%), Yb (7.15%), and Yc groups (5.55%) after 4 days, whereas no significant change was observed in groups Yd (1.97%) and Ye (1.15%) compared to the control group Wa (1.89%) (Figure 3A).

In the control group and the Yc and Yd groups, Enterobacteriaceae was the predominant family in the Wa, Yc, and Yd groups after 1–2 days of feeding. Alcaligenaceae increased significantly and became the most abundant family over time. In comparison, Pseudomonadaceae was significantly higher in the Ya and Yb groups after 1 day of feeding, accounting for 48.02 and 64.56% of the total flora, respectively. The Flavobacteriaceae in the Ya and Yb groups fed with high concentrations of *P. aeruginosa* increased significantly with larval growth and became the dominant family, followed by Enterobacteriaceae and Alcaligenaceae (Figure 3B).

**FIGURE 4:**
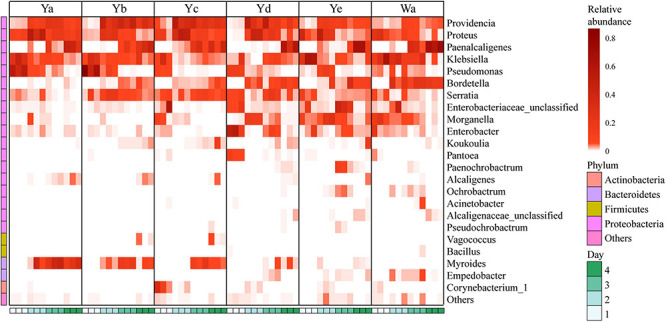
Heat maps of the relative abundances and distributions of bacterial genera in housefly larvae. The heatmap illustrates the relative percentage of each genus in each sample. The color code indicates the relative abundance, ranging from faint (low abundance) to intense (high abundance). Each treatment included three biological replicates. Wa, Ya, Yb, Yc, Yd, and Ye represent housefly larvae samples fed diets with sterile water, *P. aeruginosa* stock solution, and the stock solution diluted 10^2^, 10^4^, 10^6^, and 10^8^ fold, respectively.

A total of 24 genera were identified from all groups. *Paenalcaligenes*, *Proteus*, *Providencia*, and *Klebsiella* were the most abundant bacterial genera (accounting for 15.78 14.77, 14.67, and 12.43% of the total intestinal flora, respectively) (Figure 3C). The housefly larvae showed distinctive gut community structures after feeding on different concentrations of *P. aeruginosa*. After a high concentration of *P. aeruginosa* was fed upon, the relative abundance of *Pseudomonas* increased significantly in a short time (Ya1d and Yb1d) and then returned to normal levels (Figure 4). With the growth of larvae, the relative abundances of *Providencia*, *Proteus*, *Myroides*, *Klebsiella*, and *Alcaligenes* increased significantly (Ya3d, Yb3d, Ya4d, and Yb4d) in housefly larvae fed with high concentrations of *P. aeruginosa*, while the relative abundances of *Bordetella*, *Enterobacter*, *Morganella*, *Ochrobactrum*, *Alcaligenaceae*, and *Empedobacter* were significantly reduced (Ya3d, Yb3d, Ya4d, and Yb4d). After 4 days, the most abundant bacterial genera were *Proteus*, *Myroides*, and *Pseudomonas* (21.85, 19.87, and 14.43%, respectively) in the Ya group. The most abundant bacteria genera were *Providencia*, *Pseudomonas*, and *Paenalcaligenes* (25.20, 18.22, and 12.17%, respectively) in the Yb group. It should be noted that, compared to the control group, a low concentration of *P. aeruginosa* (Yd and Ye) in the diet had less impact on the intestinal flora of housefly larvae (Figure 4). As the concentration of *P. aeruginosa* in the housefly larvae decreased, *Providencia*, *Proteus* and *Paenalcaligenes* became the predominant genera in both the Yc (24.78, 19.54, and 13.79%, respectively) and Yd groups (18.92, 14.10, and 13.02%, respectively) after feeding for 4 days, while in the Ye group, the most abundant bacterial genera became *Klebsiella*, *Paenalcaligenes*, and *Proteus* (15.81, 14.11, and 13.59%, respectively). Compared to the control group, whose dominant genera were *Paenalcaligenes*, *Bordetella*, *Klebsiella*, and *Proteus* (22.62, 15.02, 21.60, and 9.57%, respectively), no significant change in *Paenalcaligenes* and *Proteus* was observed, with the *P. aeruginosa* concentration changing in the larval diet after feeding for 4 days. However, the bacterial abundances of *Myroides*, *Providencia*, *Pseudomonas*, *Bordetella*, *Morganella*, and *Klebsiella* showed significant differences as dilutions of *P. aeruginosa* increased in the diet (Figure 3C).

**FIGURE 5:**
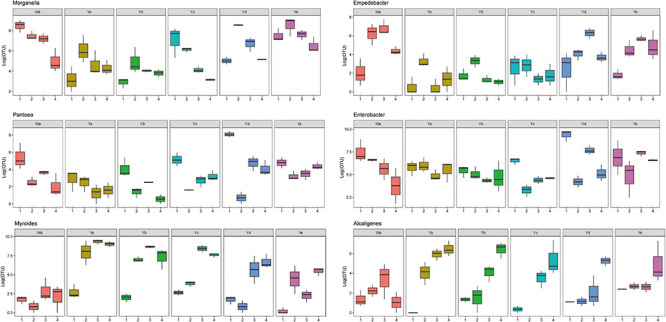
Dynamic variation of OTU number of key bacteria in different groups fed with different dilution concentrations of *P. aeruginosa*. Wa, Ya, Yb, Yc, Yd, and Ye represent housefly larvae samples fed diets with sterile water, *P. aeruginosa* stock solution, and the stock solution diluted 10^2^, 10^4^, 10^6^, and 10^8^ fold, respectively.

Based on heatmap results, we found that the compositions and structures of the intestinal microflora in houseflies fed different diets were dynamic (Figure 4). A dynamic analysis of the key bacterial genera revealed that the bacterial abundances of *Morganella*, *Empedobacter*, *Enterobacter*, and *Pantoea* decreased as the concentration of *P. aeruginosa* increased, while the bacterial abundances of *Myroides* and *Alcaligenes* increased (Figure 5). After high concentrations of *P. aeruginosa* (Ya and Yb) were fed upon, the bacterial abundances of *Paenalcaligenes* and *Bordetella* increased with increasing feeding time, and the bacterial abundance of *Klebsiella* decreased with increasing feeding time (Supplementary Figure 3).

### Effect of *P. aeruginosa* Y12 on the Community Structure of the Gut Microbiota in Housefly Larvae

**FIGURE 6:**
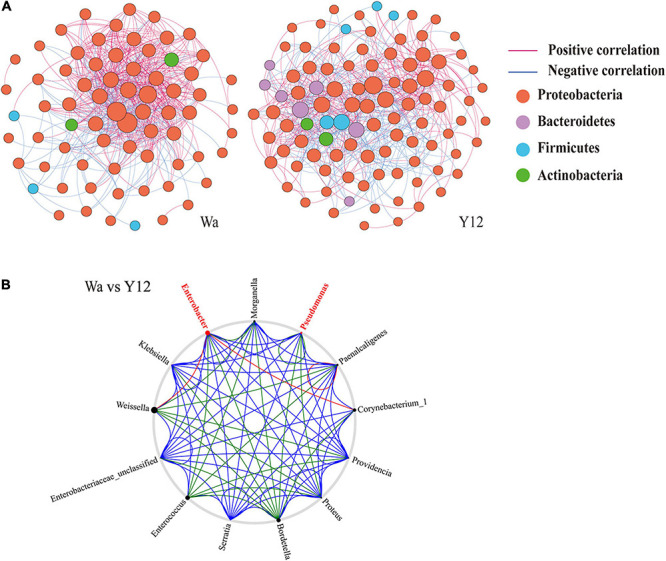
Networks **(A)** and co-occurrence networks **(B)** based on intragroup and intergroup intestinal microbiomes. **(A)** Network analysis of the control group and *P. aeruginosa* Y12 group. Each point in the figure represents a species, and species with correlations are connected by a line. Red lines represent positive correlations, blue lines represent negative correlations, and the intensity of the line represents the level of correlation. **(B)** Potential “driver taxa” of infection based on bacterial network analysis of the Y12 experimental groups and the control group W, marked as Y12-W. Node sizes are proportional to their scaled NESH (neighbor shift) score (a score identifying important microbial taxa of microbial association networks), and the nodes colored red are important driver taxa. As a result, large red nodes denote particularly important taxa driving feeding on *P. aeruginosa*. Line colors indicate node (taxa) connections as follows: association present in only experimental groups (red edges), association present in only the control group (green edges), and association present in both experimental and control groups (blue edges).

To investigate the effects of time and *P. aeruginosa* concentration on the bacterial community structure of the gut microbiota in houseflies, a correlation network of the housefly gut microbiota was constructed. We found that feeding *P. aeruginosa* significantly changed the interactions between the intestinal flora components of housefly larvae. Compared to those of the control group, the OTU nodes and edges of Bacteroidetes and Firmicutes in the intestinal flora of housefly larvae fed *P. aeruginosa* increased. The total nodes and edges in the flora interaction network also increased. The average path length was longer, while the density and average clustering coefficient were reduced. A lower level of positive connectivity was observed in the *P. aeruginosa* feeding group, while a higher negative correlation was found (Table 2). Furthermore, in the control group, three phyla, Proteobacteria, Firmicutes and Actinobacteria, were found. A high level of connectivity within Proteobacteria was observed. However, after feeding with *P. aeruginosa*, the correlation of the gut microbiota with Proteobacteria was reduced, which was followed by increasing interactions between Bacteroidetes, Firmicutes, Actinobacteria, and Proteobacteria. It should be noted that Bacteroidetes, which showed no significant interactions with the gut microbiota in the control group, revealed a significant correlation with Firmicutes, Actinobacteria, and Proteobacteria after feeding with *P. aeruginosa* (Figure 6A). In “NetShift” analysis, we linked the *Enterobacter* and *Pseudomonas* genera as potential key bacterial taxa in the initial microbiomes of houseflies fed *P. aeruginosa* (Figure 6B).

**TABLE 2:** Co-occurrence network indices of different groups.

**Network Indexes**	**Total nodes**	**Total links**	**Average path**	**Average clustering**	**Positive**	**Negative**
			**Distance**	**Coefficient**	**Correlation**	**Correlation**
Wa	71	528	2.005	0.486	70.27%	29.73%
Y12	99	584	2.34	0.285	60.62%	39.38%

*Wa and Y12 represent housefly larvae samples fed sterile water and *P. aeruginosa*. Each treatment included three biological replicates. Wa: sterile water; Y12: *P. aeruginosa*.*

### The Mutual Benefits and Competition Between *P. aeruginosa* and Other Cultivable Bacteria in the Housefly Intestine

A total of 10 cultivable bacterial species belonging to 9 genera were isolated from the intestine of housefly larvae, including *E. hormaechei, K. pneumoniae, P. aeruginosa, A. bereziniae, P. stuartii, E. cloacae, L. lactis, L. fusiformis, P. vermicola*, and *B. safensis*. To explore the influence of *P. aeruginosa* on cultivable bacteria in the housefly intestine, plate confrontation assay analysis was carried out between different bacteria (Supplementary Figure 4). We found that compared to the bacteria grown on a control agar plate, bacteria grown on an agar plate with *P. aeruginosa* revealed a significantly lower growth rate. Our results revealed that *P. aeruginosa* had an inhibitory effect on the isolated bacteria and that the growth of *E. hormaechei, K. pneumoniae, A. bereziniae, P. stuartii, E. cloacae, L. fusiformis, P. vermicola*, and *B. safensis* was arrested by *P. aeruginosa* (Table 3).

**TABLE 3:** Bacteriostatic effect of *P. aeruginosa* and other cultivable bacteria in the housefly larval intestine.

**Culturable bacteria**	**Control group/mm**	**Experimental group/mm**	***t***	***P***
*Enterobacter hormaechei*	10.33 ± 0.58	6.33 ± 0.58	8.485	0.001**
*Klebsiella pneumoniae*	10.33 ± 0.58	6.67 ± 0.58	7.778	0.002**
*Acinetobacter bereziniae*	11.67 ± 0.58	6.33 ± 0.58	11.310	0.000***
*Providencia stuartii*	11.67 ± 0.58	7.67 ± 0.58	8.485	0.001**
*Enterobacter cloacae*	10.33 ± 0.58	6.33 ± 0.58	8.485	0.001**
*Lactococcus lactis*	6.33 ± 0.58	7.00 ± 0.00	2.000	0.116
*Lysinibacillus fusiformis*	11.00 ± 1.00	6.67 ± 0.58	6.500	0.003**
*Providencia vermicola*	12.00 ± 0.00	6.67 ± 1.15	8.000	0.001**
*Bacillus safensis*	10.00 ± 0.58	6.67 ± 0.58	7.778	0.002**

Each treatment included three biological replicates. The error estimate represents the standard error of the mean. *p < 0.05, **p < 0.01, ***p < 0.001.

### Effects of the Isolated Bacteria on the Development of Housefly Larvae

**FIGURE 7:**
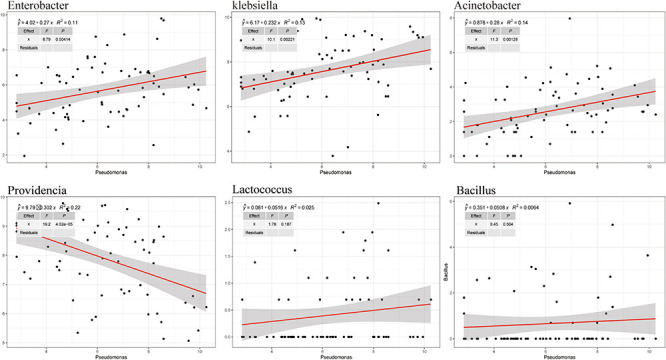
Correlation between *Pseudomonas* and other cultivable bacterial genera in housefly larvae fed isolated bacteria.

To analyze the effects of the isolated bacteria on the growth of houseflies, housefly larvae were treated by adding the isolated bacteria to their diet. We found that, compared with the control treatment, *E. hormaechei, K. pneumoniae, E. cloacae, L. fusiformis*, and *B. safensis* significantly promoted the development of housefly larvae and *A. bereziniae* had a smaller promoting effect on the larvae; However, *P. stuartii and P. vermicola* exhibited an inhibitory effect on the growth of housefly larvae in a short time; Additionally, *L. lactis* had no effect on the growth of housefly larvae (Supplementary Figure 5). Additionally, the correlations between *Pseudomonas* strains and other cultivable strains were analyzed. High-throughput sequencing analysis revealed that the entire community structure of housefly gut microbial flora varied with the OTU changes for *Pseudomonas.* Positive correlations indicated mutually beneficial relationships between *Pseudomonas* and the genera *Enterobacter*, *Acinetobacter*, and *Klebsiella*, while a negative correlation between *Pseudomonas* and *Providencia* indicated that a competitive relationship existed. Furthermore, little correlation was observed between *Pseudomonas* and *Lactococcus* and *Bacillus* (Figure 7).

## Discussion

**FIGURE 8:**
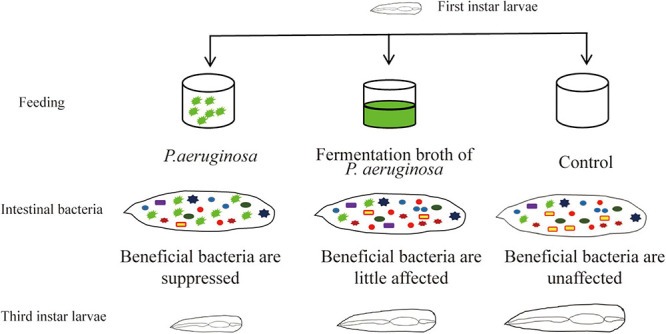
Pattern diagram of the influence *P. aeruginosa* exhibited on the growth of housefly larvae.

Gut microbial communities play an important role in the growth of insects. The symbiotic interactions between the gut microbiota and its host insect ensure healthy development of the insect. Disturbance of the gut microbiota affects host health and results in disease. Houseflies that can utilize animal manure and regenerate biofertilizer have been used as ecological biodegradation insects for animal manure management (Čičková et al., 2012; Hall et al., 2018). In addition, larvae themselves could be used as nutritional animal feed (Wang et al., 2017). However, pathogenic microorganism, especially *Pseudomonas* sp., infections affect the growth of housefly larvae and even cause death (Olcott et al., 2010; Johnson et al., 2019; Wang et al., 2020). Till now, there has been little research on the mechanism by which *P. aeruginosa* pathogenesis inhibits the development of housefly larvae. The interactions between *P. aeruginosa* and the community diversity of the gut microbiota are unknown. In this study, the mechanism by which high concentrations of *P. aeruginosa* inhibit housefly growth was studied. We analyzed the role of the gut microbiota in the interactions of the *P. aeruginosa* with its housefly hosts and the influence of *P. aeruginosa* on the microbial community structures of housefly larvae was investigated. Our results revealed that high concentrations of *P. aeruginosa* disturbed the composition of the gut microbiota and suppressed the growth of beneficial bacteria, which disrupted the intestinal barrier and therefore inhibited the development of housefly larvae (Figure 8).

The bacteria detected in this study belong to four different phyla, among which Proteobacteria was the most abundant. At the family level, Enterobacteriaceae was prominently abundant, which is consistent with previous research results for housefly larvae fed different diets (Xue et al., 2019). Enterobacteriaceae is also a dominant family in many other Tephritid species, including medfly (*C. capitata*) (Aharon et al., 2013) and citrus fruit fly (*Bactrocera minax*) (Wang A. et al., 2014). It has been reported that adding Enterobacteriaceae strains (*Klebsiella pneumonia, Enterobacter* spp*., Citrobacter freundii*, and *Klebsiella oxytoca*) to the diets of Mediterranean fruit flies improved quality control parameters and their sexual performance (Ben Ami et al., 2010; Gavriel et al., 2011; Hamden et al., 2013). A report also revealed that Enterobacteriaceae strains such as *Enterobacter* spp. and *Klebsiella oxytoca* in Mediterranean fruit flies inhibited the growth of some harmful strains, such as *P. aeruginosa* (Behar et al., 2008). Given these effects of Enterobacteriaceae, we assumed that Enterobacteriaceae would play an important role in the early development of housefly larvae. Pseudomonadaceae increased significantly in a short time in housefly larvae fed high concentrations of *P. aeruginosa*, which could be attributed to the high concentrations of *P. aeruginosa* in the larval diet. After one day, Pseudomonadaceae overproliferation was limited, and their original abundance was restored, indicating that the intestinal flora of housefly larvae was regulated by the host, which tried to maintain the intestinal balance. However, compared to that of the control group, the community structure of intestinal bacterial communities in the housefly larvae fed high concentrations of *P. aeruginosa* changed significantly with the development of housefly larvae, the relative abundance of Flavobacteriaceae increased, and that of Alcaligenaceae decreased significantly. As *Flavobacterium* sp. have been reported to be important bacterial pathogen against animals and insects (Park et al., 2011; Rochat et al., 2019). Therefore, we assume that overgrowth of pathogenetic Flavobacterium strains would have negative effects on the health of the housefly. As the gut microbiota is crucial in protecting the host from intestinal pathogen infections, the community structure changes caused by oral ingestion of *P. aeruginosa* would be the main reason for the death of housefly larvae.

We detected an increase in the bacterial load and a significant decrease in bacterial diversity in the group fed a high concentration of *P. aeruginosa* compared to the control group. At the genus level, after feeding on *P. aeruginosa*, the bacterial abundance of dominant bacteria in the intestinal tract of housefly larvae changed significantly. When a diet of *P. aeruginosa* at different concentrations was fed upon, *Paenalcaligenes, Providencia*, and *Proteus* were always in high abundance in the intestines of housefly larvae. According to previous reports, *Proteus* maintained high abundance in the intestines of housefly larvae and inhibited the growth of the pathogenic microorganisms *Salmonella Typhi* and *P. aeruginosa*, which was consistent with our results (Greenberg and Klowden, 1972). Erdmann et al. suggested that *Proteus* can protect the host from the invasion of pathogenic microorganisms and prevent wound infection (Erdmann, 1987; Stathopoulou et al., 2019). Therefore, based on the previous research and our results, we assume that gradual reductions in *P. aeruginosa* in the intestine of housefly larvae could be attributed to the function of *Proteus* in the intestine. *Providencia* species have been reported to be pathogenetic bacteria that proliferate within the fly and cause a high level of mortality among hosts (Galac and Lazzaro, 2011). Our study revealed a higher abundance of *Providencia* in housefly larvae fed a diet of *P. aeruginosa*, especially in the Ya, Yb and Yc groups, than in control larvae. Feeding experiments showed that *Providencia stuartii* and *Providencia vermicola* inhibited the development of housefly larvae, which further revealed the important role *Providencia* played in the death of houseflies. Furthermore, *Myroides* emerged in high abundance, while *Bordetella* was in low abundance in the gut of houseflies fed high concentrations of *P. aeruginosa* compared to the control group. Antimicrobial studies have revealed that biosurfactants produced by *Myroides* can inhibit the growth of various bacteria (Dharne et al., 2008); they are also considered a cause of surgical wound and urinary tract infections, meningitis, and ventriculitis (Yağci et al., 2000). We assume that dysbiosis of the gut microbiota in the housefly fed with high-concentration *P. aeruginosa* might be caused by the antimicrobial compounds produced by *Myroides*.

The insect gut microbiota, whose members dynamically interact with each other, is a central regulator of host metabolism. The composition and function of animal intestinal flora are dynamic, and interactions between different bacterial strains play important roles in insect health and disease. Abnormal proliferation of some pathogenic strains disturbs the balance of the gut microbiota and leads to disease. Based on a network analysis, we found that oral administration of *P. aeruginosa* significantly affected the interaction network of gut flora. Higher levels of connectivity between *Firmicutes, Actinobacteria*, and *Bacteroidetes* were observed in housefly larvae fed high concentrations of *P. aeruginosa*. Furthermore, as previously described, a *Myroides* strain isolated from the gut of adult flesh flies showed antibacterial action against most bacterial strains (Dharne et al., 2008). In this study, we found that *Myroides* and *Proteus*, which belong to *Bacteroidetes*, exhibited higher bacterial abundance. Therefore, based on the structural changes in microbial networks and the differential abundances observed in our study, we assume that *P. aeruginosa* induced overgrowth of some pathogenic strains and downregulated the beneficial bacteria in the gut microbial community, which altered the balanced interaction network among the microbiota and accelerated the death of housefly larvae. Overall, we found that time and the concentration of *P. aeruginosa* are important factors that cause changes in the community composition in the intestinal microbiota of housefly larvae. This is of great significance for understanding how the complex microbial community in the gut of housefly larvae responds to invasive bacteria and other disturbances. Additionally, we suggest that the community composition of the gut microbiota plays a vital role in the health of host organisms.

Studies have reported the influence of cultivable bacteria in the intestine of housefly larvae on larval development, but there are few studies on the interactions between bacteria. In this study, a plate confrontation experiment between *P. aeruginosa* and other cultivable bacteria revealed that *P. aeruginosa* inhibited the growth of most isolated bacteria and showed antagonistic action against them. However, *E. hormaechei, K. pneumoniae, P. stuartii, E. cloacae, L. lactis, L. fusiformis, P. vermicola*, and *B. safensis*, emerged in higher abundances in the housefly larvae fed with *P. aeruginosa*. Therefore, we suggest that even though some cultivable bacteria have an antagonistic relationship with *P. aeruginosa*, the antagonism might be minimal or even mutually beneficial in the intestinal environment of the host. The bacterial species might have a competitive relationship in the natural environment, but in the gut microenvironment of housefly larvae, the interaction between *P. aeruginosa* and the other bacteria would be reduced.

Our study revealed that high concentrations of *P. aeruginosa* have a significant inhibitory effect on housefly larvae. Oral administration of *P. aeruginosa*, especially high concentrations of *P. aeruginosa*, resulted in an abnormal increase or decrease of specific bacteria. We assume that the abnormal increase of enteropathogenic strains and decrease of beneficial strains would contribute to the disorder of gut microbiota. Administration of *P. aeruginosa* changed the bacterial diversity of the intestinal microflora, influenced the interactions between the entire intestinal networks and induced a high level of mortality. As a balanced gut microbiota contributes to the health of the host, alteration of the community structure of the gut microbiota would result in disease in the insect. Additionally, we assume that the virulence factors produced by *P. aeruginosa* could have some negative effects on the host insect. We would analyze the influence of the virulence factors to the insect health in our further research.

In conclusion, our results illustrated the important role of the housefly gut microbiota after *P. aeruginosa* infections and provided evidence that a high concentration of *P. aeruginosa* induced dysbiosis of the gut microbiota, which provides insight for further study of the pathogenic mechanism of *P. aeruginosa* in housefly larvae.

## Data Availability Statement

The raw data supporting the conclusions of this article will be made available by the authors, without undue reservation.

## Author Contributions

QZ: methodology, formal analysis, software, investigation, data curation, writing original draft, and visualization. SW: methodology, formal analysis, investigation, and writing original draft. XZ: methodology, data curation, and visualization. RZ: investigation, formal analysis, supervision, and validation. ZZ: conceptualization, supervision, validation, review and editing, and funding acquisition. All authors contributed to the article and approved the submitted version.

## Conflict of Interest

The authors declare that the research was conducted in the absence of any commercial or financial relationships that could be construed as a potential conflict of interest.

## Footnotes

1 http://www.usadellab.org/cms/index.php?page=trimmomatic

2 https://mothur.org/

3 https://web.rniapps.net/netshift
