# Supplementary material for: Negative Impact of Pseudomonas aeruginosa Y12 on Its Host Musca domestica
Source: Front Microbiol. 2021 Jul 14;12:691158. doi: 10.3389/fmicb.2021.691158 (PMC8317488; doi:10.3389/fmicb.2021.691158)
Supplement: Supplementary file 1 [file Image_1.PDF]

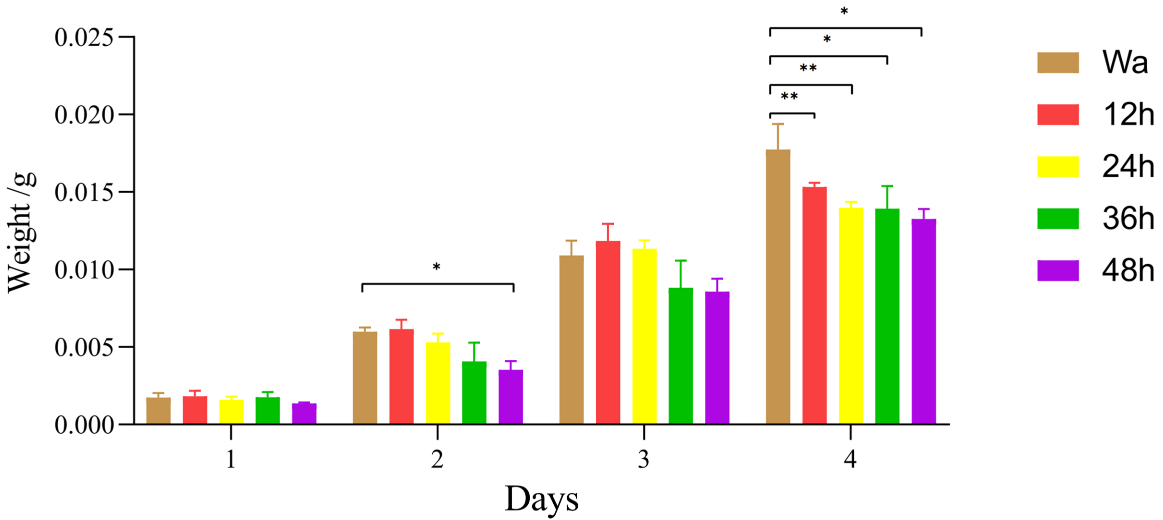

Supplementary Figure S1 Effects of the fermentation broth of *P. aeruginosa* at different times on the growth and development of housefly larvae. Wa: sterile water. Data were analysed using one-way ANOVA. Significance analysis was performed by Tukey's test. Each treatment included three biological replicates. Error bars denote standard error of the mean. *: $p < 0.05$, **: $p < 0.01$.
