# Supplementary material for: Negative Impact of Pseudomonas aeruginosa Y12 on Its Host Musca domestica
Source: Front Microbiol. 2021 Jul 14;12:691158. doi: 10.3389/fmicb.2021.691158 (PMC8317488; doi:10.3389/fmicb.2021.691158)
Supplement: Supplementary file 2 [file Image_2.PDF]

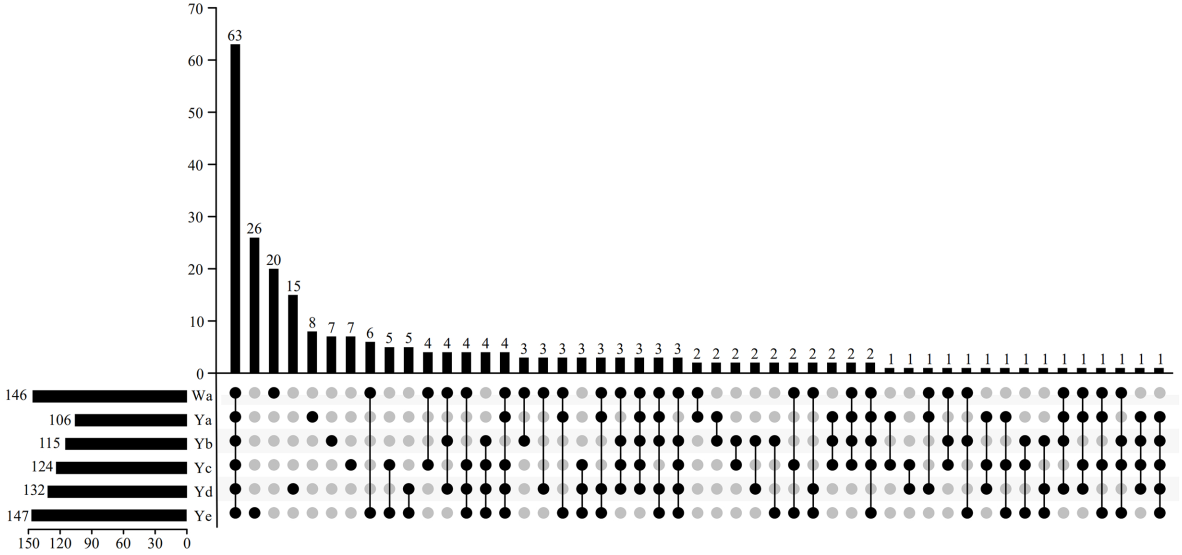

Supplementary Figure S2 Shared and unique OTU analysis of the intestinal bacteria in housefly larval samples fed different dilutions of *P. aeruginosa*, shown as a Venn diagram. Wa, Ya, Yb, Yc, Yd and Ye represent housefly larvae samples fed diets with sterile water, *P. aeruginosa* stock solution, and the stock solution diluted 102, 104, 106, and 108 fold, respectively. The numbers represent the numbers of unique OTUs in each sample and common OTUs shared by two or more samples.
