# Supplementary material for: Negative Impact of Pseudomonas aeruginosa Y12 on Its Host Musca domestica
Source: Front Microbiol. 2021 Jul 14;12:691158. doi: 10.3389/fmicb.2021.691158 (PMC8317488; doi:10.3389/fmicb.2021.691158)
Supplement: Supplementary file 3 [file Image_3.PDF]

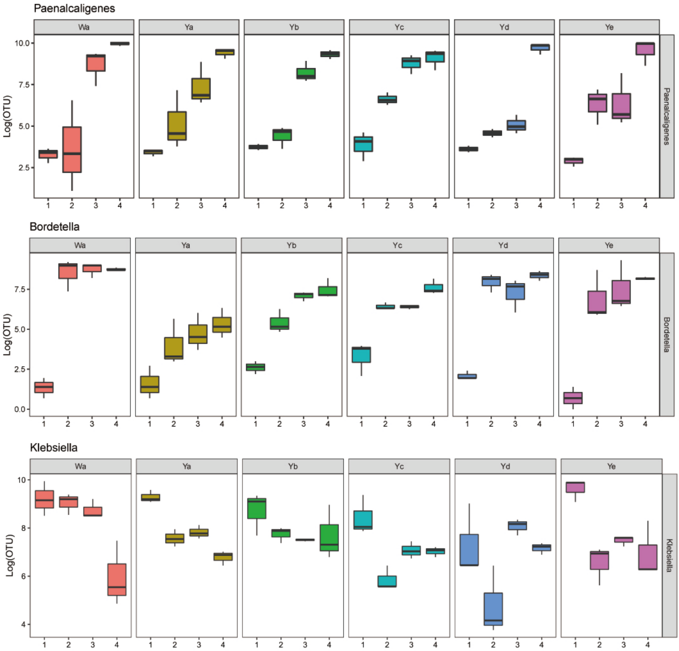

Supplementary Figure S3 Dynamic changes in the OTU number of key bacteria in different groups with the development of housefly larvae. Wa, Ya, Yb, Yc, Yd and Ye represent housefly larvae samples fed diets with sterile water, *P. aeruginosa* stock solution, and the stock solution diluted 102, 104, 106, and 108 fold, respectively.
