# Supplementary material for: Negative Impact of Pseudomonas aeruginosa Y12 on Its Host Musca domestica
Source: Front Microbiol. 2021 Jul 14;12:691158. doi: 10.3389/fmicb.2021.691158 (PMC8317488; doi:10.3389/fmicb.2021.691158)
Supplement: Supplementary file 4 [file Image_4.PDF]

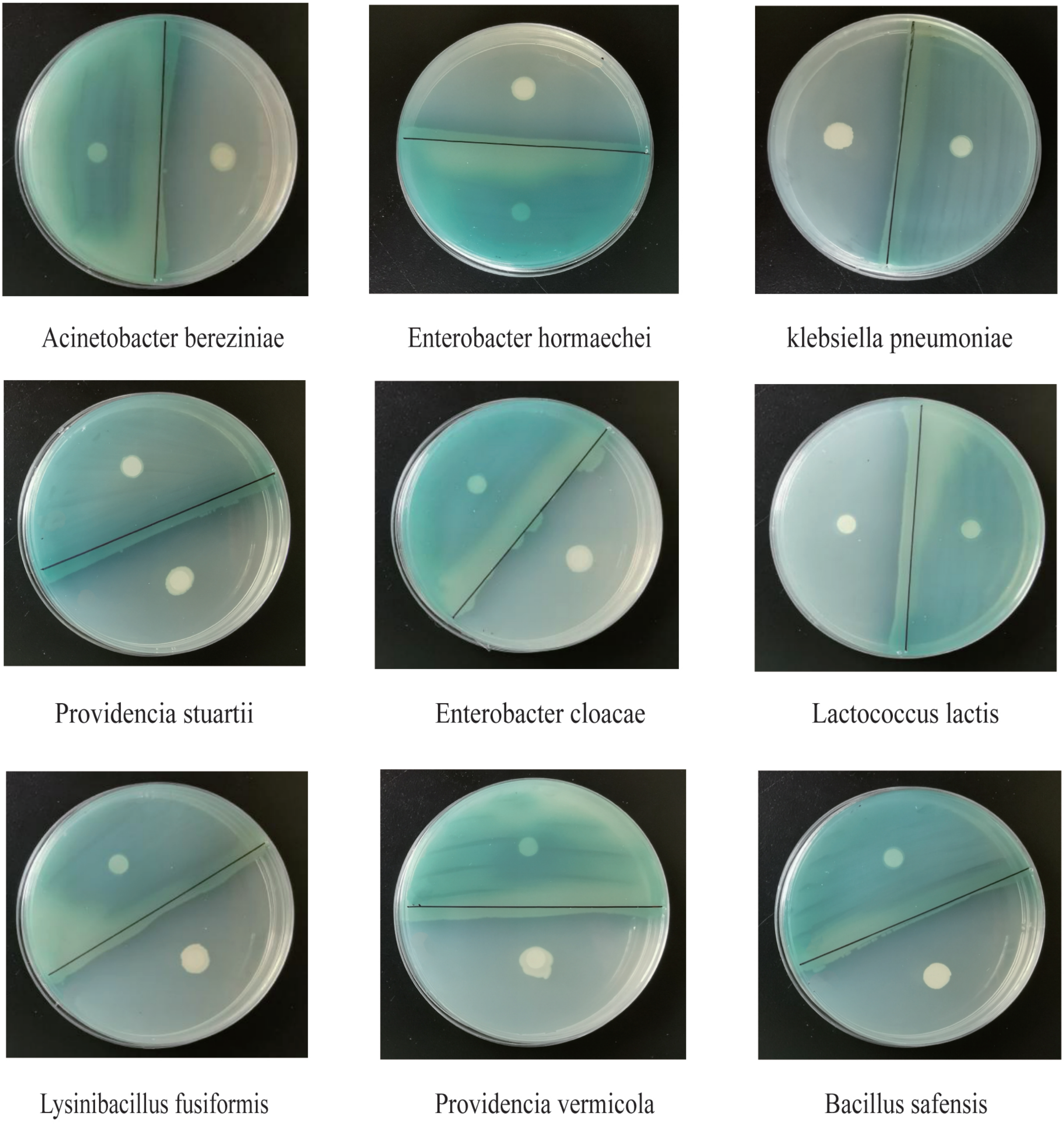

Supplementary Figure S4 Antagonistic experiment of *P. aeruginosa* and other cultivable bacteria in the housefly larval intestine.
