# Supplementary material for: Negative Impact of Pseudomonas aeruginosa Y12 on Its Host Musca domestica
Source: Front Microbiol. 2021 Jul 14;12:691158. doi: 10.3389/fmicb.2021.691158 (PMC8317488; doi:10.3389/fmicb.2021.691158)
Supplement: Supplementary file 5 [file Image_5.PDF]

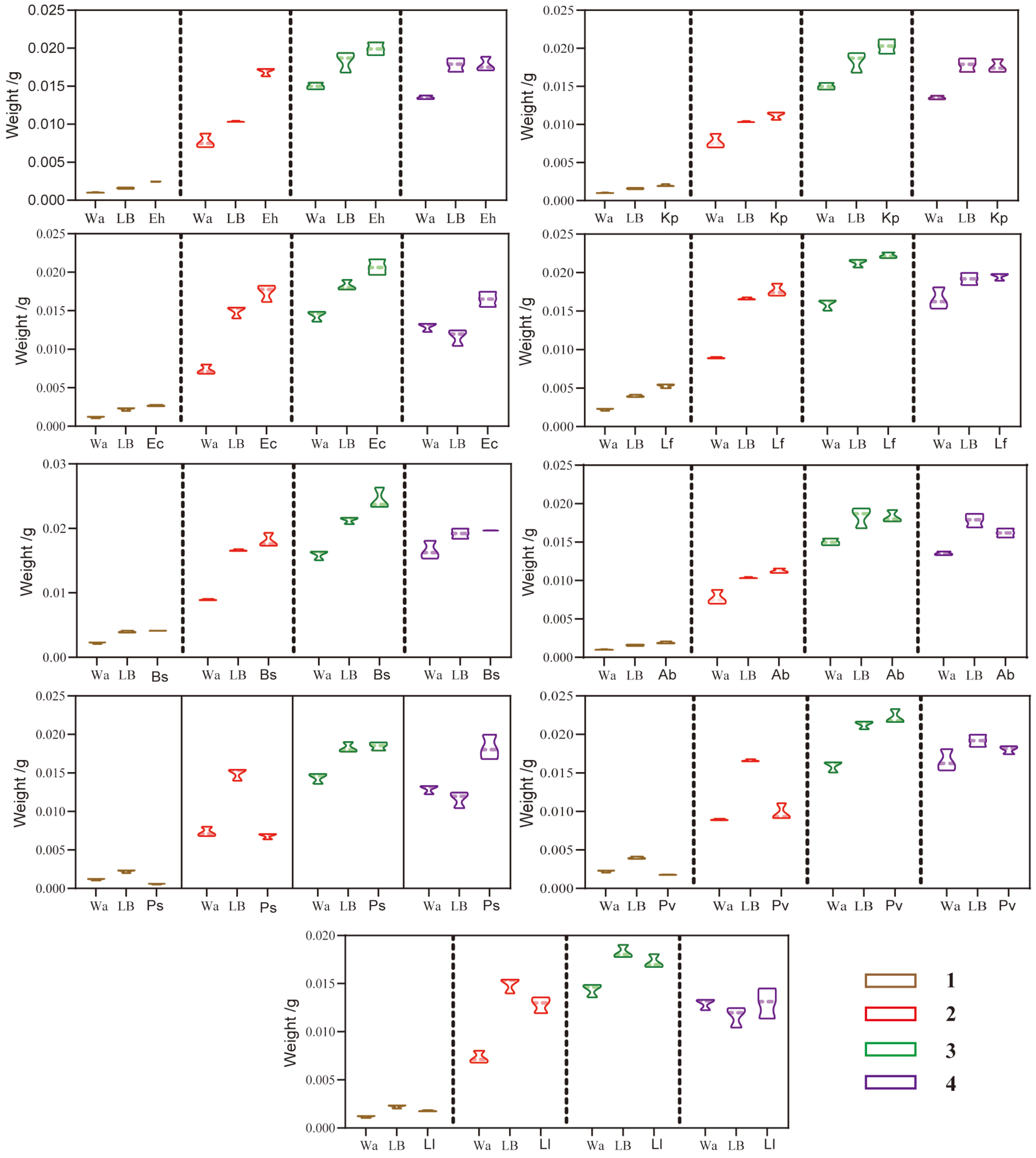

Supplementary Figure S5 Effects of other cultivable bacteria in the housefly larval intestine on larval development. Wa: sterile water; LB: liquid medium; Eh, Kp, Ab, Ps, Ec, Ll, Lf, Pv and Bs represent *Enterobacter hormaechei*, *Klebsiella pneumoniae*, *Acinetobacter bereziniae*, *Providencia stuartii*, *Enterobacter cloacae*, *Lactococcus lactis*, *Lysinibacillus fusiformis*, *Providencia vermicola* and *Bacillus safensis*, respectively.
